# Making Use of Existing International Legal Mechanisms to Manage the Global Antimicrobial Commons: Identifying Legal Hooks and Institutional Mandates

**DOI:** 10.1007/s10728-020-00393-y

**Published:** 2020-03-31

**Authors:** Susan Rogers Van Katwyk, Isaac Weldon, Alberto Giubilini, Claas Kirchhelle, Mark Harrison, Angela McLean, Julian Savulescu, Steven J. Hoffman

**Affiliations:** 1 Global Strategy Lab, Dahdaleh Institute for Global Health Research, Faculty of Health and Osgoode Hall Law School, York University, 4700 Keele Street, Dahdaleh Building 2120, Toronto, ON M3J 1P3 Canada; 2 School of Epidemiology and Public Health, Faculty of Medicine, University of Ottawa, Ottawa, Canada; 3 Department of Politics, York University, Toronto, Canada; 4 Oxford Martin School, University of Oxford, Oxford, UK; 5 Wellcome Centre for Ethics and Humanities, Oxford, UK; 6 Wellcome Unit for the History of Medicine, University of Oxford, Oxford, UK; 7 Department of Zoology, University of Oxford, Oxford, UK; 8 Oxford Uehiro Centre for Practical Ethics, University of Oxford, Oxford, UK; 9 Department of Global Health and Population, Harvard T H Chan School of Public Health, Harvard University, Boston, MA USA; 10 Department of Health Research Methods, Evidence, and Impact and McMaster Health Forum, McMaster University, Hamilton, Canada

**Keywords:** Antimicrobial resistance, International law, Global health policy, Collective action

## Abstract

Antimicrobial resistance (AMR) is an urgent threat to global public health and development. Mitigating this threat requires substantial short-term action on key AMR priorities. While international legal agreements are the strongest mechanism for ensuring collaboration among countries, negotiating new international agreements can be a slow process. In the second article in this special issue, we consider whether harnessing existing international legal agreements offers an opportunity to increase collective action on AMR goals in the short-term. We highlight ten AMR priorities and several strategies for achieving these goals using existing “legal hooks” that draw on elements of international environmental, trade and health laws governing related matters that could be used as they exist or revised to include AMR. We also consider the institutional mandates of international authorities to highlight areas where additional steps could be taken on AMR without constitutional changes. Overall, we identify 37 possible mechanisms to strengthen AMR governance using the International Health Regulations, the Agreement on the Application of Sanitary and Phytosanitary Measures, the Agreement on Trade-Related Aspects of Intellectual Property Rights, the Agreement on Technical Barriers to Trade, the International Convention on the Harmonized Commodity Description and Coding System, and the Basel, Rotterdam, and Stockholm conventions. Although we identify many shorter-term opportunities for addressing AMR using existing legal hooks, none of these options are capable of comprehensively addressing all global governance challenges related to AMR, such that they should be pursued simultaneously with longer-term approaches including a dedicated international legal agreement on AMR.

## Introduction

Antimicrobial resistance (AMR) poses an urgent threat to global public health and development. Actors at all levels—consumers, prescribers, countries, and international agencies—need to move quickly if we are to mitigate the threat posed by AMR. Reconciling this urgent need for global action on AMR with the slow pace of international law-making is one of the main challenges to crafting a global governance system that can effectively, fairly and feasibly manage the antimicrobial commons.

Legally binding international agreements represent the strongest formal mechanisms through which countries can make commitments to each other [7]. A grand bargain on AMR—a single, comprehensive, and legally binding One Health agreement—would likely be the most effective way of achieving a global governance system that can bring about ambitious AMR goals [8, 12, 18]. Such an agreement could most effectively balance the competing needs across human, animal, agriculture and environmental sectors to ensure access to antimicrobials, conservation of antimicrobial effectiveness, and innovation in antimicrobial therapies and alternative technologies.

Negotiating a complex international legal agreement on this vast scale, however, requires significant political mobilization and substantial financial commitments. The level of investment needed for global health treaties has rarely been forthcoming. Substantial international leadership is required to convene countries for negotiations, and substantial costs are associated with negotiating and maintaining treaty governance structures [7]. Developing an international legal agreement on AMR would also require substantial time investments; for example, the idea for a Framework Convention on Tobacco Control was proposed at the World Health Assembly in 1995, but formal negotiations did not begin until 1999, and the treaty did not come into legal force until 2005 [13]. Therefore, while a dedicated international legal agreement on AMR is important, valuable, and worth pursuing to address AMR over the longer-term, the international community cannot wait 10 years before beginning to collectively address the challenges posed by AMR. Shorter-term approaches must be pursued simultaneously with longer-term approaches. Rates of AMR are growing rapidly, and given the potential consequences for human life [14] and international development [26], swift and substantial global collaboration and cooperation is needed to deliver on key AMR priorities.

International law, however, may still offer valuable opportunities in the short-term, even without a grand bargain or broader longer-term legal agreement that comprehensively addresses all drivers and dimensions of AMR. International law is a valuable tool which can provide a regulatory framework that makes country commitments transparent, provides accountability for fulfilling those commitments, and disincentivizes deviation. The far-reaching nature of AMR and its root social causes mean that action is needed in domains that are already governed by existing international laws and institutions. In this article, we identify opportunities for strengthening the global governance of AMR through existing channels using “legal hooks” in various international agreements or using the structures within mandates of existing international organizations. We do this analysis by developing a list of ten key global actions needed to address AMR and then surveying the existing legal landscape for existing mechanisms through which these actions could be pursued. Overall, we identified 37 opportunities for acting on AMR using existing international legal mechanisms.

## Legal Hooks

One consequence of the complex and far-reaching nature of AMR is that facets of the problem fall into domains already governed by international law. These domains could provide legal hooks for AMR regulations, wherein existing legal provisions or principles governing closely related matters can be used either as they exist or revised to address part of what is needed for AMR. These legal hooks include existing legally binding commitments between state parties in international law that authorize necessary AMR actions or that have mandates within which new AMR-specific provisions could easily be added. For example: the International Health Regulations (IHR) already require state parties to take rapid action to detect and respond to public health risks; the World Trade Organization (WTO) Agreement on the Application of Sanitary and Phytosanitary Measures (SPS) already establishes regulations on food safety and animal and plant health standards for WTO members; and the Basel, Rotterdam, and Stockholm conventions already govern the use, trade, and disposal of hazardous chemicals, products, and wastes. After excluding regional bodies, including some very impressive developments in European Union legal frameworks on veterinary medicinal products [4], market authorization [2], and counterfeit medicines [1], we identified eight international laws in health, trade, and environmental spheres that provide opportunities for strengthening action on AMR (Table 1). These existing agreements offer opportunities to address AMR using existing legal powers, or opportunities to revise the scope and powers of the agreements to specifically address AMR.

**Table 1:** Existing international legal instruments with potential links to AMR

Treaty	Abbreviation
International Health Regulations (2005): An international legal agreement through the World Health Organization that promotes global health security by building capacity to detect, assess, report and respond to public health risks	IHR
Agreement on the Application of Sanitary and Phytosanitary Measures (1995): An international legal agreement under the World Trade Organization to support the right of governments to protect food safety, plant and animal health, and prevent these sanitary and phytosanitary measures from being unjustified trade barriers	SPS
Agreement on Trade-Related Aspects of Intellectual Property Rights (1995): An international legal agreement between members of the World Trade Organization which sets minimum standards for the regulation of intellectual property by national governments	TRIPS
Agreement on Technical Barriers to Trade (1995): An international legal agreement through the World Trade Organization that aims to ensure that technical regulations and standards are non-discriminatory and do not create unnecessary obstacles to trade	TBT
International Convention on the Harmonized Commodity Description and Coding System (1983): An international legal agreement under the World Customs Organization to facilitate trade and information exchange by harmonizing the description, classification, and coding of goods in international trade, including controlled products	HCDCS
Basel Convention on the Control of Transboundary Movements of Hazardous Wastes and Their Disposal (1989): An international legal agreement that limits harmful waste pollution, promotes environmentally sound management of hazardous wastes, and restricts the transboundary movements of hazardous wastes	Basel
Rotterdam Convention on the Prior Informed Consent Procedure for Certain Hazardous Chemicals and Pesticides in International Trade (1998): An international legal agreement designed to protect human health and the environment from chemicals that remain intact in the environment, become widely distributed geographically, accumulate in the fatty tissue of humans and wildlife, and have harmful impacts on human health or on the environment	Rotterdam
Stockholm Convention on Persistent Organic Pollutants (2001): An international legal agreement designed to protect human health and the environment by eliminating or restricting the production and use of persistent organic pollutants	Stockholm

## Institutional Mandates

Additionally, as part of their respective constitutions, many international organizations—including the World Health Organization (WHO), United Nations Food and Agriculture Organization (FAO), and United Nations Environment Program (UNEP)—have powers to create international legal agreements. Specifically, both WHO and FAO’s constitutions (Articles 19 and 14 respectively) allow their plenary decision-making bodies (the World Health Assembly and the Conference of the FAO, respectively) to adopt agreements that become legally binding on their membership after a two-thirds majority vote and subsequent ratification at the domestic level [5, 23]. The United Nations Environmental Assembly, the UN’s highest-level decision-making body on the environment, also provides a forum through which environmental treaties can be negotiated.

One important limitation is that each international organization is restricted to making legal agreements that are consistent with their own constitutional mandates. A broad agreement that addresses all drivers and dimensions of AMR would likely need to transcend these sectoral divisions and require immense collaboration between these bodies; however, given their powers to convene countries and recommend international policy, these agencies are in a position—individually or collaboratively—to support the creation of legally binding obligations within their specific mandate areas. Each of these technical agencies, along with the World Organization for Animal Health (OIE), have substantial powers to collect and interpret scientific data, recommend national and international actions, and provide funding and technical assistance to achieve global aims within their particular domains. Leading non-governmental organizations can also take important steps towards addressing AMR by mobilizing awareness, interest, and action. Additionally, the Global Fund to Fight AIDS, Tuberculosis & Malaria, Gavi, and UNITAID could incorporate AMR into their portfolios.

## Strengthening AMR Responses Through Existing International Legal Mechanisms

To explore opportunities for addressing AMR through existing legal mechanisms, we developed a list of ten key global actions to address AMR across access, conservation and innovation. To improve *access* to antimicrobials, action is required to: (1) develop equitable pricing and licensing models to support access to antimicrobials in resource limited settings; (2) end the manufacturing, sale and export of substandard, falsified and banned antimicrobial products; and (3) strengthen health systems to improve infection prevention and control and reduce the need for antimicrobials. On the *conservation* front, more concerted international action is needed to: (4) strengthen surveillance of antimicrobial use and antimicrobial resistance in humans, animals and the environment; (5) promote the responsible use of antimicrobials in humans; (6) promote the responsible use of antimicrobials in animals and agriculture; (7) safeguard the effectiveness of newly developed antimicrobials; and (8) limit antimicrobial contamination in the environment. For innovation, global action is needed to: (9) incentivize the development of new antimicrobials and related technologies; and (10) facilitate the development of new antimicrobials and related technologies (Table 2).

**Table 2:** Possible legal hooks and institutional mandates for addressing ten key issues in antimicrobial resistance

Access						
	Key global actions on AMR	Possible legal hooks				
1	Develop equitable pricing and licensing models to support access to antimicrobials in resource limited settings	*TRIPS* Authorize compulsory licenses for generic antimicrobials for those resource limited setting that have effective stewardship regimes in place	*IHR* Create a global pooled fund that would equitably support appropriate access to antimicrobials, the conservation of these medicines, and infection prevention initiatives	*GAVI/Global Fund* Participate in a global procurement mechanism that minimizes the costs of existing antimicrobials and related technologies	*TRIPS/TBT* Implement a global tiered pricing system and mitigate parallel importation so that antimicrobials and related technologies can be sold at lower prices in lower-income countries without diminishing markets in higher-income countries	*TBT* Strategically use tariffs and tax policy to make antimicrobials less expensive as appropriate for countries where antimicrobials are currently underutilized
2	End the manufacturing, sale and export of substandard, falsified and banned antimicrobial products	*TBT* Ensure monitoring and compliance with rigorous quality assurance standards for antimicrobials	*HCDCS* Enforce compliance with rigorous quality assurance standards for antimicrobials at time of import/export	*WHO/FAO/OIE* Require surveillance of substandard and falsified antimicrobials, and education about the harms of using these products		
3	Strengthen health systems to improve infection prevention and control and reduce the need for antimicrobials	*IHR* Provide financial and technical support for all countries to achieve core public health capacities and universal health coverage	*IHR* Create and finance a global fund to improve access, stewardship, health care, food security, and water, sanitation and hygiene systems			

Conservation						
	Key global actions on AMR	Possible legal hooks				
4	Strengthen the surveillance of antimicrobial usage and AMR in humans, animals and the environment	*IHR* Set international standards for laboratory testing, antimicrobial usage and AMR surveillance, and data harmonization in humans, animals, and the environment	*IHR or WHO/FAO/OIE mandate* Designate an international expert authority to develop antimicrobial usage guidelines for humans, animals, and the environment	*IHR or WHO/FAO/OIE mandate* Develop national and regional authorities on antimicrobial stewardship, drug standards, and AMR surveillance to report back to the international expert authority	*IHR or WHO/OIE/FAO* Make available technical expertise to strengthen AMR and antimicrobial usage surveillance infrastructures	*SPS* Provide funding for strengthening monitoring and surveillance of antimicrobial usage/AMR in LMIC through the Standards and Trade Development Facility
5	Promote the responsible use of antimicrobials in humans	*TBT/SPS/IHR/WHO* Ban commercial advertising and marketing of antimicrobials	*WHO mandate* Require a prescription from an authorized health worker for all antimicrobial use in humans	*WHO mandate* Regulate prescribers to delink any provision of antimicrobials from their financial remuneration		
6	Promote the responsible use of antimicrobials in animals and agriculture	*SPS or FAO mandate* Ban the use of antimicrobials for growth promotion in animals	*SPS or FAO mandate* Limit prophylactic antimicrobial use to single animals	*SPS or FAO* Ban the metaphylactic use of antimicrobials in animal production	*TBT or FAO mandate* Require a prescription from an authorized animal health worker for all antimicrobial use in animals and ban cascade prescribing	*FAO mandate* Regulate prescribers to delink any provision of antimicrobials from their financial remuneration
7	Safeguard the effectiveness of new antimicrobials	*IHR or WHO/FAO mandate* Create an expert committee to monitor resistance and, as needed, designate and reserve particular antimicrobials for human-only use	*TRIPS/TBT or WHO mandate* Automatically reserve new antimicrobial classes for exclusive human usage	*TBT* Strategically use tariffs and tax policy to make antimicrobials more expensive as appropriate for countries where antimicrobials are currently overutilized	*TRIPS/TBT* Prevent the manufacturing, sale and export of new antimicrobial classes for animal use until authorized by a designated international entity	
8	Limit antimicrobial contamination in the environment	*Basel, Rotterdam, Stockholm* Set, monitor and enforce national guidelines for antimicrobial waste management in the environment	*UNEP* Develop international regulations on the disposal of antimicrobial waste	*UNEP* Make available technical expertise to limit antimicrobial contamination in the environment		

Innovation						
	Key global actions on AMR	Possible legal hooks				
9	Incentivize the development of new antimicrobials and related technologies	*IHR* Require national investments in R&D for new antimicrobials, alternative therapeutics, diagnostics, social interventions and infection prevention that are proportional to national economic capacity and antimicrobial use	*UNITAID or GARDP* Experiment with innovative financing mechanisms for R&D needed to address AMR	*WHO/global fund* Participate in a global pooled fund that would equitably support open research in those countries that have achieved progress towards conserving antimicrobials		
10	Facilitate the development of new antimicrobials and related technologies	*WHO/FAO/OIE/UNEP* Create a process to develop product profiles and designate social outcomes for new technologies or approaches that should be prioritized	*TBT/international conference on harmonization* Expedite the assessment and regulatory approval of prospective new antimicrobials and related technologies	*WHO* Report annually on all investments in antimicrobial R&D		

### Access to Antimicrobials

Banning the manufacture, sale and export of substandard and falsified drugs is a prime opportunity for addressing AMR access concerns through international law. These substandard and falsified drugs are a growing global problem that can cause morbidity and mortality. Substandard drugs have not passed the usual standard and quality testing protocols and include falsified medicines that have been deliberately mislabeled with respect to their origin or contents [9]. A specific ban on such drugs might entail regulations on trade through the WTO’s Agreement on Technical Barriers to Trade (TBT) that require and ensure monitoring and compliance with rigorous quality assurance standards for antimicrobials. Similarly, the International Convention on the Harmonized Commodity Description and Coding System (HCDCS), with its focus on customs, could enforce compliance with these quality standards at the time of import and export. Finally, the WHO, FAO and OIE could support efforts to end the manufacturing and sale of these products through increased surveillance and education with regards to the harms associated with these products.

Another way to improve access to safe and effective medicine is through patent licensing [15]. Under the WTO's Agreement on Trade-Related Aspects of Intellectual Property Rights (TRIPS), governments can allow someone besides the patent holder (including the government itself) to produce a patent-protected product or use a patent-protected process for their domestic market. In normal circumstances, governments can attempt to obtain a voluntary license, which is a voluntary arrangement between the patent holder and government. In the event where a voluntary agreement cannot be reached, governments can issue a compulsory license which permits them to license the patent without the permission of the patent holder, provided that they first attempted to obtain a voluntary license. In extenuating circumstances, like in settings without the domestic capability of manufacturing high quality medicines, governments can issue a compulsory license of a patent and arrange for manufacturers in other countries to make the product for them, again provided that they first attempted to obtain a voluntary agreement [27]. These mechanisms, already authorized by international trade law, can increase global accessibility of antimicrobials. While licensing is an effective way to procure drugs at a low cost, it requires additional action to offset its potentially disruptive impact on innovation. In order to balance the tension between affordable drugs today and investment by the pharmaceutical industry in new drugs for the future, licensing must be paired with creative funding mechanisms such as prizes for newly developed technologies or a global fund to compensate patent holders when governments need to license antimicrobials.

### Conservation

International law could be a powerful tool to address the continuing rise of non-human antimicrobial use. A ban on non-therapeutic antimicrobial use through the FAO, or by employing regulations on trade through the SPS, could substantially enhance global antimicrobial stewardship. Either mechanism could include a minimum requirement of a universal ban of antimicrobial growth promotion and could also require parties to phase out prophylactic and metaphylactic non-human use of all antimicrobials by a certain year, such as 2030. Although the impact on human health remains imprecise, the non-human use of antimicrobials vastly exceeds global human use and has historically repeatedly selected for resistance factors that have impacted human health [10, 11, 22]. Protecting human health and acknowledging the One Health dimensions of AMR entails a stringent reduction of non-essential antimicrobial usage in global food production and major improvements in antibiotic stewardship in human health and the environment. Similarly, revisions could be made to the IHR or TRIPS to prospectively, automatically and strictly limit any new class of antimicrobials to human use only, until a designated international organization like the WHO, FAO or OIE has determined that its non-human use is safe for humans. Other measures will also help reduce non-human antimicrobial usage. Bans on the use of antimicrobials for animal growth promotion in agriculture have already been enacted in the EU, US, and other countries. Further restrictions on the use of critically important antimicrobials to treat entire herds (“metaphylaxis”) or medicate animals before infections are present (“prophylaxis”) were agreed by the European Parliament in 2018 [3]. Both growth promoter bans and restrictions on meta- and prophylaxis are designed to preserve antimicrobial effectiveness by limiting their non-human use and prioritizing essential therapeutic usage among those humans and animals who really need them.

Revisions to the TBT, SPS, and IHR could also be made to promote appropriate antimicrobial use such as by banning the commercial advertisement, marketing, and sales promotion of antimicrobials. These international efforts could reduce misinformation about antimicrobial effectiveness, dampen the transnational spread of this misinformation through social media, and strengthen efforts to curb the substantial international grey and black markets supplying substandard and falsified antimicrobials.

Beyond legal hooks, the mandates of various international organizations with the power to create international laws could be used to promote conservation goals. A WHO treaty could start by protecting human health and end with a call to transition away from using critically important antimicrobials in agriculture, and an FAO treaty could start with a call to transition away from critically important antimicrobials and end with a focus on developing, promoting, and supporting sustainable agricultural practices. Such treaties might emanate from Article 19 or 21 of the WHO’s constitution and Article 14 of the FAO’s constitution respectively, engage with WHO’s AWaRe framework, create provisions to limit the agricultural use of critically important antimicrobials for human health, and set standards to protect any new antimicrobials for future human health needs. In addition to being feasible within the existing legal mandates of the WHO and FAO, at least one large pharmaceutical company has committed not to license new antimicrobials for animal use [6], demonstrating there may be support for such rules from unexpected places.

A third possible avenue could be to strengthen surveillance and data sharing by adopting new legally binding regulations with existing institutional powers. New international standards are also required to standardize antimicrobial usage data collection, which should include statutory national reporting requirements of domestic and international sales data by commercial antimicrobial producers and distributors. A global surveillance infrastructure has to be politically neutral, transparent, and overseen by appropriate technical organizations. Ultimately these data must be gathered in a way that elicits trust. To avoid underreporting and geographic biases, the international community will probably have to establish and finance an integrated network of independent AMR reference laboratories around the world. At present there are a limited number of countries participating in WHO’s GLASS program as a result of data sharing concerns, different standards for data collection and reporting, different definitions of AMR, and limited data collection capacity in some countries [17, 24]. Given the importance of high-quality global surveillance data to track new resistance threats, it may be possible to develop an international body that provides funding and expertise for strengthening surveillance, while creating mandatory reporting and data sharing standards. Such a focused agreement could make use of Article 21 of WHO’s constitution, which allows for the development of new legally binding regulations without requiring ratification from its 194 member states through their regular national processes, because it falls within scope of the enumerated matters on which such regulations are allowed [16]. That being said, due to the multisectoral nature of the surveillance problem, a more focused treaty would still benefit from drawing upon the expertise of the WHO-FAO-OIE tripartite plus UNEP, instead of just one technical agency. This presents an alternative option: the United Nations General Assembly (UNGA) could adopt an AMR surveillance treaty using the joint enterprise method that delegates technical authority to the tripartite. This method would avoid regularly bringing contentious AMR issues to UNGA, increasing the potential to reach a consensus without risking ambition.

### Innovation

International law presents several opportunities to address the existing governance and market failures that have led to insufficient levels of antimicrobial innovation and to incentivize and facilitate the development of new antimicrobials and related technologies. The IHR, for example, could be revised to require national investments in research and development for new antimicrobials, alternative therapeutics, diagnostics, social interventions and infection prevention approaches that are proportional to national economic capacity and antimicrobial use. Existing legal mechanisms can also explore new and innovative funding models. In fact, some new and promising government and joint public–private partnerships have already arisen in response to the global challenge of restoring the pipeline for new antimicrobials [19]. But drug development has many stages and support is needed at all of them. In the absence of an overarching governance framework for funding AMR-related innovations, a disproportionate amount of money has been poured into basic scientific research in the form of early-stage push funding with fewer resources available for other types of needed research and at other stages of the innovation pipeline. This distribution, while a good start, can still lead to stagnation if discoveries in these early stages are not shepherded along the pipeline to later stages. Other later-stage push and pull funding mechanisms designed to bring drugs through clinical trial and into the marketplace are critically needed, but are lacking because of an absence of funding and governance [19]. Another viable alternative may be to consider fully public development models for drugs [20]. Better coordination on how innovation is funded is needed. A global pooled fund or institution governed by a single entity likely represents the best solution and can be housed as a joint enterprise between the WHO, FAO and OIE, and possibly managed by the Global Fund to Fight AIDS, Tuberculosis & Malaria.

Legal mechanisms can also be used to facilitate the development of new antimicrobials and their alternatives. At the very least, a dynamic list of all AMR-related research and development needs can be compiled and published by WHO, FAO, OIE and UNEP which could help to reduce inefficient duplications [21]. Such a mechanisms could not only develop product profiles, but also designate social outcomes for new technologies and approaches. Second, international law could address limitations in the way that new drugs are reviewed and approved around the world. Market authorization can be a long and costly process that diminishes the net potential value of new drugs, which further diminishes prospects for private investment in antimicrobial innovation [15]. Expedited review processes and internationally harmonized regulations under the TBT and International Conference on Harmonization could help. For example, if a new antimicrobial can be quickly approved in one country and then be subsequently and simply approved in other countries thereafter, it would significantly remove regulatory barriers that prevent new drugs from coming to market.

## Discussion

Overall, we have identified 37 possible mechanisms to strengthen AMR governance in the short-term using existing legal hooks and structures. All of these mechanisms have the theoretical potential to expedite AMR action by focusing on narrowly defined issues and amending or employing elements of existing agreements. In many cases, these mechanisms also offer an opportunity to engage with actors that do not usually participate in AMR policymaking. However, adapting existing international law to meet AMR challenges also presents several practical challenges, among which are a lack of appetite for revising existing international agreements, the proliferation of uncoordinated global AMR actions, and the difficulty of consolidating enough expertise on every treaty’s unique procedures and norms while also ensuring enough future expertise on AMR in those governing bodies. For these reasons, we suspect that efforts to use amend existing law to create short-term action on AMR will be more fragmented, less focused, and ultimately less successful than long-term efforts to create an enduring legal agreement for AMR.

Future attempts to curb AMR should be comprehensive in scope and coordinated at the international level. But significant challenges exist when creating responses beyond the national level. The most pressing of these challenges are prioritization, inequality, implementation, and metrics [12]. AMR goals will only be achieved through concerted and coordinated international action, yet smaller treaties may result in a more fragmented, less coordinated response that does not adequately or equitably address access, conservation and innovation goals. Coordinating the creation and subsequent governance of all AMR-specific provisions could potentially lead to inefficient or ineffective duplications and loopholes. For example, existing standards for AMR surveillance are already out of step between human and animal sectors [25], and substantial work will be needed to ensure that these do not continue to diverge. Finally, it is also important to acknowledge that addressing AMR through existing legal mechanisms is likely to result in inequitable burdens and outcomes based on gender, ethnicity, wealth, geography and other considerations. Ensuring that these challenges are acknowledged and addressed may prove more difficult when working within existing legal mechanisms than when developing a new, fit-for-purpose agreement.

Although AMR is a One Health problem—and the WHO-FAO-OIE tripartite has taken admirable steps to ensure that it is seen as such—many countries continue to see AMR exclusively as a human health issue. This leads to AMR being addressed separately from animal, agricultural, environmental and trade issues. Successfully using the various international legal mechanisms we have identified above will require breaking down these silos and ensuring that officials in other sectors recognize AMR as being sufficiently important for their attention, action and leadership. The absence of a clear linear correlation between antimicrobial usage and AMR burdens means that regulatory interventions will have to go beyond simply addressing antimicrobial consumption, yet communicating these challenges outside health and veterinary fields remains difficult.

## Conclusion

Every use of an antimicrobial can theoretically select for resistant organisms and resistance genes, with repercussions for global AMR. Reducing antimicrobial use across the human health, animal and agricultural sectors will thus reduce but not eliminate the likelihood of AMR proliferation. As a “silver bullet” solution to AMR is unlikely to ever exist, it is important to protect antimicrobial effectiveness using the strongest available tools for international commitment and cooperation. While an all-encompassing treaty would likely be best for ensuring equity, minimizing duplication of efforts, and avoiding loopholes that threaten the common pool of antimicrobial effectiveness, negotiating such a grand bargain could take several years. Substantial action is required in the short-term to safeguard human health and development. The idea of negotiating a series of revisions to existing international legal agreements is not incompatible with the idea of a single comprehensive grand bargain and, in the short-term, may permit quicker progress on specific dimensions of AMR that in turn help to build momentum for greater progress thereafter. Making revisions to several existing treaties may not be as efficient or elegant as a single treaty fully encompassing of a grand bargain, however, pursuing some, or several, of these strategies in parallel could promote continued and increasing international cooperation on AMR, and offer opportunities to increase meaningful One Health cooperation with actors situated outside the traditional AMR sphere.
